# Pulmonary tuberculosis in Brazilian indians: a picture of this context depicted through radiography

**DOI:** 10.1590/0100-3984.2015.48.5e1

**Published:** 2015

**Authors:** Marcos Duarte Guimarães

**Affiliations:** 1 PhD, Supervisor of Medical Residency in Imaging Diagnosis at Hospital Heliópolis, Professor, Program of Post-graduation stricto sensu, A.C.Camargo Cancer Center, São Paulo, SP, Brazil. E-mail: marcosduarte500@gmail.com.

Currently the Brazilian indigenous population is estimated to be a little more than 817 thousand individuals scattered all over the regions of the country, according to the 2010 demographic census. Such a population is the remainder of the original five-million indigenous population that lived in the Brazilian forests at the time of the Europeans' arrival. They are found in greater number in the North and Central-West regions where about 90% of the demarcated lands are located, with 60% of the population distributed principally throughout the states of Roraima, Amazonas and Mato Grosso do Sul^(1)^.

Unfortunately, there are no reliable data about the true health conditions of the Brazilian indigenous population. The data made available by some official organs such as Fundação Nacional do Índio (FUNAI) (National Indian Foundation), Fundação Nacional de Saúde (FUNASA) (National Health Foundation), Secretaria Especial de Saúde Indígena (SESAI) (Special Secretary of Indigenous Health) and non-governmental organizations demonstrate high rates of morbidity and mortality in the indigenous population, frequently superior to those observed in the general Brazilian population^(2,3)^. Such a reality is a result from intrinsic immunological characteristics, living habits, malnutrition and elevated rates of infectious diseases such as gastrointestinal infections, respiratory infections, tuberculosis (TB), sexually transmissible diseases and malaria^(2)^.

The occurrence of tuberculosis (TB) is closely related to a low index of human development (IHD) characterized by unfavorable social conditions, low schooling index, and precarious housing and health conditions - unfortunately a sad reality present in most indigenous villages of our country. Available data indicate that, amongst indigenous individuals, the tuberculosis incidence rate in 2013 (95.6/100,000 individuals) was three times higher than the incidence rate observed in the general population for the same period (35.4/100,000 individuals)^(2,4)^. The data from SESAI regarding mortality are not less alarming. Such data indicate that respiratory diseases, particularly the infectious ones, were responsible for 15.3% of the total number of indigenous individuals deaths recorded in that same year, about two times the mortality rate for those diseases in the general population^(2-4)^.

Those indicators might be improved with the implementation of actions of basic attention to health involving the indigenous population. Strengthening of the sanitary coverage, improvement of the accessibility to health centers, screening for infectious diseases, therapeutic surveillance on an outpatient basis, and an active search for the individuals who abandon the treatment represent measures that would be capable of reducing the severe consequences of this disease^(1-4)^. In this context, studies like the one developed by Lachi and Nakayama, and published in the present issue of **Radiologia Brasileira**, can bring some contribution to a better understanding about TB-specific aspects in indigenous populations, thus allowing for a more appropriate diagnostic approach and a better therapeutic management^(5)^. In the mentioned study, the authors have analyzed radiographic findings in the chest of 81 indigenous individuals treated for TB in a hospital in the city of Dourados, state of Mato Grosso do Sul, Brazil. It is interesting to observe that almost all (97.5%) the images presented some finding such as consolidation, nodule, pleural involvement, excavation, fibrosis, lymph node enlargement, atelectasis and calcifications. Amongst such examinations, 61.7% presented at least three areas affected by the disease, inferring a remarkable extent and severity of the findings. Another interesting finding is that almost 42% of the affected individuals in this population were aged under 30, differently from what occurs in the general Brazilian population whose mean age of affected individuals is between 45 and 49 years^(3,5)^.

Chest radiography is recommended for screening for TB in populations at risk such as workers in health services, prisoners, individuals living in nursing homes, shelters, or indigenous villages^(6)^. It has several advantages for being a simple, low-cost and easy-toperform imaging method that exposes the patient to low ionizing radiation doses, capable of detecting several alterations suggestive of TB and widely available in health services, even in those of lower complexity^(7)^. In certain populations, such as prisoners, chest radiography is recommended as an initial method of screening for TB, even in the absence of respiratory symptoms^(8)^. Exceptionally, computed tomography may be employed in cases where bacilloscopy and chest radiography cannot confirm the diagnosis in individuals with respiratory symptoms^(6)^.

For all the above reasons, I invite the reader to read the article and understand that by means of chest radiography it is possible to recognize the main findings of pulmonary tuberculosis and contextualize its severity in the Brazilian indigenous population that lacks minimal health conditions to survive, maintaining their cultures and traditions.
